# A novel signature to predict the neoadjuvant chemotherapy response of bladder carcinoma: Results from a territory multicenter real-world study

**DOI:** 10.3389/fgene.2022.1047481

**Published:** 2022-11-02

**Authors:** Huihuang Li, Jiao Hu, Xiongbing Zu, Minfeng Chen, Jinbo Chen, Yihua Zou, Ruoping Deng, Gang Qin, Wenze Li, Jiansheng Tang, Dingshan Deng, Jinhui Liu, Chunliang Cheng, Yu Cui, Zhenyu Ou

**Affiliations:** ^1^ Department of Urology, Xiangya Hospital, Central South University, Changsha, China; ^2^ National Clinical Research Center for Geriatric Disorders, Xiangya Hospital, Central South University, Changsha, China; ^3^ Department of Urology, The First People’s Hospital of Chenzhou, Chenzhou, China; ^4^ Department of Urology, The Central Hospital of Yongzhou, Yongzhou, China; ^5^ Department of Urology, The First People’s Hospital of Xiangtan City, Xiangtan, China; ^6^ Department of Urology, Affiliated Hospital of Xiangnan University, Chenzhou, China

**Keywords:** bladder carcinoma, neoadjuvant chemotherapy, pathological response, personalized therapy, risk score

## Abstract

**Background:** Although neoadjuvant chemotherapy (NAC) has become the standard treatment option for muscle invasive bladder carcinoma (MIBC), its application is still limited because of the lack of biomarkers for NAC prediction.

**Methods:** We conducted a territory multicenter real-world study to summarize NAC practice in China and its associated clinicopathologic variables with NAC response. Then, we developed and validated a robust gene-based signature for accurate NAC prediction using weighted correlation network analysis (WGCNA), the least absolute shrinkage and selector operation (LASSO) algorithm, a multivariable binary logistic regression model, and immunohistochemistry (IHC).

**Results:** In total, we collected 69 consecutive MIBC patients treated with NAC from four clinical centers. The application of NAC in the real world was relatively safe, with only two grade Ⅳ and seven grade Ⅲ AEs and no treatment-related deaths being reported. Among these patients, 16 patients gave up surgery after NAC, leaving 53 patients for further analysis. We divided them into pathological response and non-response groups and found that there were more patients with a higher grade and stage in the non-response group. Patients with a pathological response could benefit from a significant overall survival (OS) improvement. In addition, univariate and multivariate logistic analyses indicated that tumor grade and clinical T stage were both independent factors for predicting NAC response. Importantly, we developed and validated a five-gene-based risk score for extremely high predictive accuracy for NAC response.

**Conclusion:** NAC was relatively safe and could significantly improve OS for MIBC patients in the real-world practice. Our five-gene-based risk score could guide personalized therapy and promote the application of NAC.

## Introduction

Bladder carcinoma (BLCA) is one of the most commonly diagnosed carcinomas and a major cause of death globally (Siegel et al., 2019). For muscle invasive bladder carcinoma (MIBC), cisplatin-based neoadjuvant chemotherapy (NAC) plus radical cystectomy (RC) is becoming the standard treatment option (Witjes et al., 2020). Several randomized clinical trials (RCTs) have demonstrated that NAC significantly increased the overall survival (OS) rates compared to RC alone, and this result was also supported by a meta-analysis (Grossman et al., 2003; Advanced Bladder Cancer Meta-analysis Collaboration, 2005; Griffiths et al., 2011; Kitamura et al., 2014). Specifically, patients who achieve a pathologic response (downstaging to ≤ pT1 at cystectomy) after NAC have a strong trend of better OS and disease-specific survival (DSS) (Grossman et al., 2003). However, not all MIBC patients could benefit from NAC, and this fact largely limits the application of NAC (Rosenblatt et al., 2012). In fact, only 30–40% of patients can achieve a pathologic response after NAC, and the remaining patients have even worse survival outcomes than RC alone (Bhindi et al., 2017; Lyon et al., 2019). To avoid chemotherapy-related toxicity and delay radical surgery, identifying which patients could benefit from NAC is vital for the treatment of MIBC.

There are some available reports about biomarkers predictive of response to NAC in MIBC. The most commonly reported is the association between mutation of DNA damage repair (DDR) genes and response to NAC, including *ATM*, *RB1*, *FANCC*, *ERCC2*, and *FGFR3* (Plimack et al., 2015; Liu et al., 2016; Yang et al., 2018; Motterle et al., 2020). Molecular subtypes of MIBC are also commonly reported. Basal tumors are characterized by aggressiveness and a better response to NAC, while p53-like tumors are characterized by resistance to NAC (Choi et al., 2014). Other biomarkers include a 12- and a 14-gene-based prediction scoring system developed by a small number of patients (Takata et al., 2005; Kato et al., 2011). However, none of these reports have been translated into clinical applications partly because of low predictive accuracy and complex detection methods. In this study, we conducted a retrospective collection from multicenter databases of patients treated with NAC and summarized their clinicopathologic features and chemotherapy-related toxicity. Importantly, we developed and validated an NAC prediction scoring system for convenient clinical application.

## Materials and methods

### Patient selection

We conducted a retrospective collection from multicenter databases for consecutive MIBC patients treated with NAC from 2017 to 2021. Ethical approval was obtained from each center, including Xiangya Hospital, the First People’s Hospital of Chenzhou, the Central Hospital of Yongzhou, and the First People’s Hospital of Xiangtan City. All the patients included had histologically proven bladder carcinoma and clinical stage T2-T4 N0-2 M0 disease and were then followed for at least two cycles of NAC. We obtained written informed consent from each patient included and registered this study on the Chinese Clinical Trial Registry (http://www.chictr.org.cn/index.aspx), registration number (ChiCTR2100047632).

### Treatment choices

Chemotherapy regimens and number of cycles were administered based on clinical practice and guidelines at the discretion of clinicians. Specifically, most patients received three cycles of cisplatin and gemcitabine. Day 1: cisplatin (70 mg/m^2^) and gemcitabine (1,000 mg/m^2^) administered intravenously (IV). Day 8: gemcitabine (1,000 mg/m^2^) IV; this was repeated every 3 weeks. For patients with renal insufficiency (creatinine clearance <60 ml/min), carboplatin-based therapy was administered. After NAC cycles, all patients were recommended to undergo RC and lymphadenectomy unless they refused strongly. In addition, there were some patients who responded well to NAC only receiving transurethral resection of bladder tumor (TURBT) because of a strong willingness of bladder sparing. For these patients, complete TURBT and radiography were performed to confirm non-muscle invasiveness. Urinary diversions after RC included ileal conduit and cutaneous ureterostomy. The decision on which diversion was to be used was mainly based on patients’ performance status (PS) and the preferences of the patients and surgeons.

### Outcome measurement

Response to NAC was defined based on the pathological stage at RC. Patients without residual MIBC (downstaging to ≤ pT1) were defined as having a pathological response, while the remaining patients were defined as having a pathological non-response (≥pT2). For patients who received TURBT only, we regarded all of them as pathological responses after confirming non-muscle invasiveness by complete TURBT and radiography. Chemotherapy-related toxicity was assessed using the acute and subacute toxicities of the anti-cancer drug indexing table (WHO). OS was calculated from the initial day of NAC administration to death from any cause. We censored patients at the last follow-up time, 1 December 2021.

### Sources of public databases

GSE69795 (GPL14951) (McConkey et al., 2016) and GSE52219 (GPL14951) (Choi et al., 2014) are two public datasets containing MIBC patients treated with NAC. We downloaded these two datasets using the “GEOquery” R package and transformed the gene symbols using the corresponding platform “GPL14951.” There were 61 samples in GSE69795, of which only 38 patients had detailed response information. We excluded the remaining 23 patients and took the 38 patients for further analysis. For GSE52219, there were 23 patients with detailed response information, and we included all of them for analysis.

### Coexpression module networks

We used the “WGCNA” R package to generate a coexpression module network and selected the gene modules with the closest relationship with NAC therapy response. As reported in our previous study (Li et al., 2021), we first filtered out the bad genes and samples using microarray data from GSE69795. Then, we calculated the connection strength and built a scale-free network based on the filtered genes and samples. We set the degree of independence as 0.85 and chose the most suitable soft power value. We developed scale-free gene coexpression networks based on the selected soft power value and selected the module that had the closest relationship with NAC therapy response. The genes in this module were selected to build a risk score for predicting NAC response.

### Immunohistochemistry and scoring

Pretreatment formalin-fixed paraffin-embedded (FFPE) MIBC tissues were collected from the Department of Pathology, Xiangya Hospital, and then, IHC was conducted, as described in our previous study (Hu et al., 2020). The anti-CEP83 antibody (PA5-113541, Invitrogen) was used. The H-score system, which integrates the staining intensity and percentage of positive cells, was adopted to evaluate the IHC score (Budwit-Novotny et al., 1986). For staining intensity, a score of 0 indicated no staining, a score of 1 indicated weak staining (faint yellow), a score of 2 indicated moderate staining (pale brown), and a score of 3 indicated strong staining (brown). For the percentage of positive cells, a score of 1 for samples with <25% positive cells, a score of 2 for samples with 25–49% positive cells, a score of 3 for samples with 50–74% positive cells, and a score of 4 for samples with ≥75% positive cells were determined. Then, the IHC score was determined by multiplying the intensity score and the percentage score. Two independent pathologists reviewed the IHCs.

### Statistical analysis

We expressed the continuous variables as the mean (range) and compared them by t-test or the Mann‒Whitney U test. Dichotomous variables were compared using Pearson’s chi-squared test or Fisher’s exact test implemented in the “gmodels” R package. The significance of each clinical variable for NAC response was assessed using univariate logistic regression analysis, and only the variables associated with NAC response with a p-value less than 0.1 were included for multivariate analysis. Both univariate and multivariate logistic regression analyses were conducted using the “glm” function. Survival curves were compared using the log-rank test and plotted using the Kaplan‒Meier method. We used the least absolute shrinkage and selector operation (LASSO) algorithm to further narrow down the genes selected in the weighted correlation network analysis (WGCNA) step. We then developed a risk score for predicting NAC response using the multivariable binary logistic regression model in the “glm” function: risk score = Σβі*RNAi. The predictive accuracy for NAC response was quantified using receiver operating characteristic (ROC) curves implemented in the “pROC” R package. *p* < 0.05 was regarded as statistically significant, and all analyses were two-sided and conducted by R software (4.0.3).

## Results

### Patient characteristics

To show the real-world practice of NAC for MIBC from 2017 to 2021 in China, we collected all 70 consecutive MIBC patients treated with chemotherapy before RC from Xiangya Hospital, the First People’s Hospital of Chenzhou, the Central Hospital of Yongzhou, and the First People’s Hospital of Xiangtan City. One patient received only one cycle of NAC and was excluded, leaving 69 patients for further analysis. None of these patients received adjuvant therapy. The clinicopathologic variables of all the patients are reported in Supplementary Table S1. All patients were in good PS with an Eastern Cooperative Oncology Group (ECOG) score of 0 or 1. Most of the patients were pathologically diagnosed with pure urothelial carcinoma (92.8%), while the remaining patients had mixed tumors (7.25%), including urothelial carcinoma mixed with squamous and sarcomatoid carcinoma cells. The clinical T stage of all the patients ranged from T2 to T4. For patients with clinical N stage of N1 and N2, we rearranged them as clinical lymph node-positive patients (N+, 15.9%). Two patients received an NAC program of gemcitabine and carboplatin because of renal insufficiency, while all of the remaining patients received a cisplatin-based NAC program. All patients were recommended to undergo RC and lymphadenectomy after NAC. Unfortunately, 16 patients (23.2%) gave up treatment because of economic reasons or a lack of confidence in curing. Eight patients received TURBT (11.6%) or partial cystectomy (1.45%) because of a strong willingness of bladder sparing.

**FIGURE 1:**
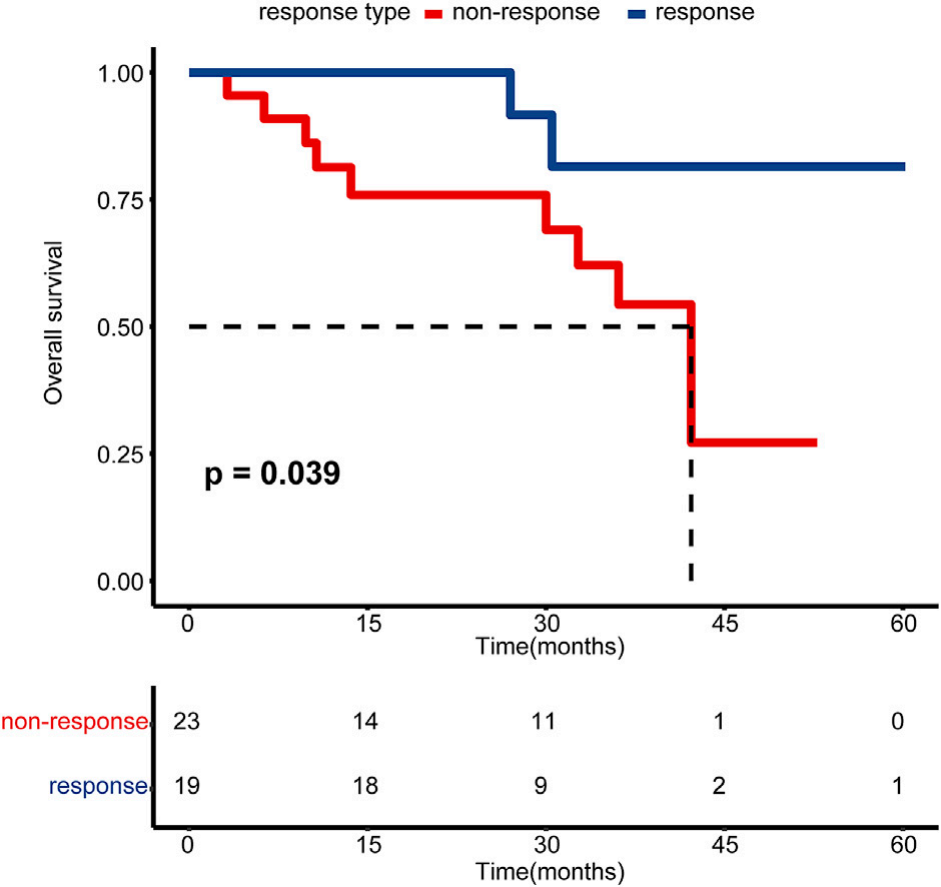
Kaplan–Meier estimated plot comparing the overall survival of patients with pathological response and non-response groups. The red line represents the non-responsive group, while the blue line represents the responsive group.

The primary objective of this study was pathological response. We defined patients without residual MIBC (downstaging to ≤ pT1) as having a pathological response (response group), while the remaining patients (≥pT2) were defined as having a pathological non-response (non-response group). For patients who received TURBT only, we regarded all of them as pathological responses after confirming non-muscle invasiveness by complete TURBT and radiography. For one patient who received partial cystectomy, we included him into a non-response group based on persistent muscle invasiveness at partial cystectomy. As shown in Table 1, there were 25 patients in the NAC response group and 28 patients in the NAC non-response group. Among these 53 patients, 42 patients were successfully followed up. Patients in the response group exhibited a significantly higher overall survival outcome than those in the non-response group (Figure 1, *p* = 0.039). There was no significant difference between these two groups for the majority of clinicopathologic variables, including age, sex, body mass index (BMI), smoking status, history of hematuresis, history of non-muscle invasive bladder carcinoma (NMIBC), hydronephrosis, the neutrophil-to-lymphocyte ratio (NLR), the pathological type, and N stage (Table 1). For pathological grade and clinical T stage, there were significantly more patients with higher grade (85.7% vs. 60.0%, *p* = 0.03) and stage (85.7% vs. 44.0%, *p* = 0.001) in the NAC non-response group (Table 1).

**TABLE 1:** Comparison of baseline clinicopathologic variables between neoadjuvant chemotherapy (NAC) response and non-response groups.

	Responder, *n* = 25	Non-responder, *n* = 28	p-value
Age (years), median (range)	60.0 (30–80)	62.3 (45–79)	0.42
Gender, n (%)	—	—	0.10
Male	24 (96.0%)	22 (78.6%)	—
Female	1 (4.00%)	6 (21.4%)	—
BMI (kg/m2), median (range)	22.5 (16–28.2)	22.0 (15.2–28.9)	0.57
Smoking status, n (%)	—	—	0.10
Non-smokers	7 (28.0%)	14 (50.0%)	—
Smokers	18 (72.0%)	14 (50.0%)	—
Hematuresis, n (%)	—	—	0.10
No	1 (4.00%)	6 (21.4%)	—
Yes	24 (96.0%)	22 (78.6%)	—
History of NMIBC, n (%)	—	—	1.00
No	21 (84.0%)	23 (82.1%)	—
Yes	4 (16.0%)	5 (17.9%)	—
Hydronephrosis, n (%)	—	—	0.26
No	18 (72.0%)	16 (57.1%)	—
Yes	7 (28.0%)	12 (42.9%)	—
NLR, median (range)	3.53 (1.20–11.5)	3.33 (1.10–9.18)	0.76
Pathological type, n (%)	—	—	0.49
Pure urothelial carcinoma	25 (100%)	26 (92.9%)	—
Mixed tumors	0 (0%)	2 (7.14%)	—
Pathological grade	—	—	0.03
Low	10 (40.0%)	4 (14.3%)	—
High	15 (60.0%)	24 (85.7%)	—
T stage	—	—	0.001
T2	14 (56.0%)	4 (14.3%)	—
T3/T4	11 (44.0%)	24 (85.7%)	—
N stage	—	—	0.47
N0	22 (88.0%)	22 (78.6%)	—
N+	3 (12.0%)	6 (21.4%)	—

BMI, body mass index; NMIBC, non-muscle invasive bladder carcinoma; NLR, neutrophil-to-lymphocyte ratio; mixed tumors, urothelial carcinoma mixed with squamous and sarcomatoid carcinoma cells.

### Treatment and safety

Supplementary Table S2 shows the summarized adverse events (AEs) of all the patients. For grade Ⅲ–Ⅳ events, only two patients reported grade IV anemia, and five and two patients reported grade Ⅲ anemia and decreased white blood cells, respectively. There were no other grade Ⅲ–Ⅳ events reported. Other grade Ⅰ–Ⅱ AEs could recover after simple symptomatic treatment, and no patient delayed surgery because of these AEs.

### Pretreatment factors for predicting NAC response

The results of univariate and multivariate logistic analysis results are reported in Table 2. The results are presented as odds ratios (ORs, 95% CIs). All the baseline clinicopathologic variables were included in univariate logistic analysis except the pathological type because there were no patients with mixed tumors in the NAC response group (Table 1). All the remaining variables except pathological grade and clinical T stage had no significant association with NAC response. Higher grade (0.25, 95% CI 0.06–0.89, *p* = 0.04) and clinical T stage (0.13, 95% CI 0.03–0.46, *p* = 0.003) were significantly associated with NAC non-response. In addition, higher grade (0.14, 95% CI 0.02–0.69, *p* = 0.03) and clinical T stage (0.16, 95% CI 0.03–0.61, *p* = 0.01) remained independent factors in multivariate logistic analysis (Table 2).

**TABLE 2:** Univariate and multivariate logistic analysis results of variables associated with neoadjuvant chemotherapy (NAC) response.

	Univariate		Multivariate	
Variable	OR (95% CI)	p-value	OR (95% CI)	p-value
Age (years)	0.98 (0.92–1.03)	0.45	—	—
Gender				
Female	Reference	—	Reference	—
Male	6.55 (1.01–128.8)	0.09	11.6 (0.91–406.1)	0.10
BMI	1.06 (0.88–1.28)	0.56	—	—
Smoking status				
Non-smokers	Reference	—	—	—
Smokers	2.57 (0.84–8.44)	0.11	—	—
Hematuresis				
No	Reference	—	Reference	—
Yes	6.55 (1.01–128.8)	0.09	7.36 (0.56–288.7)	0.20
History of NMIBC				
No	Reference	—	—	—
Yes	0.88 (0.19–3.74)	0.86	—	—
Hydronephrosis				
No	Reference	—	—	—
Yes	0.52 (0.16–1.61)	0.26	—	—
NLR	1.04 (0.81–1.34)	0.75	—	—
Pathological grade				
Low	Reference	—	Reference	—
High	0.25 (0.06–0.89)	0.04	0.14 (0.02–0.69)	0.03
T stage				
T2	Reference	—	Reference	—
T3/T4	0.13 (0.03–0.46)	0.003	0.16 (0.03–0.61)	0.01
N stage				
N0	Reference	—	—	—
N+	0.50 (0.10–2.15)	0.37	—	—

OR, odds ratio; CI, confidence interval; BMI, body mass index; NMIBC, non-muscle invasive bladder carcinoma; NLR, neutrophil-to-lymphocyte ratio.

### Development and validation of an NAC-predicting risk score

**FIGURE 2:**
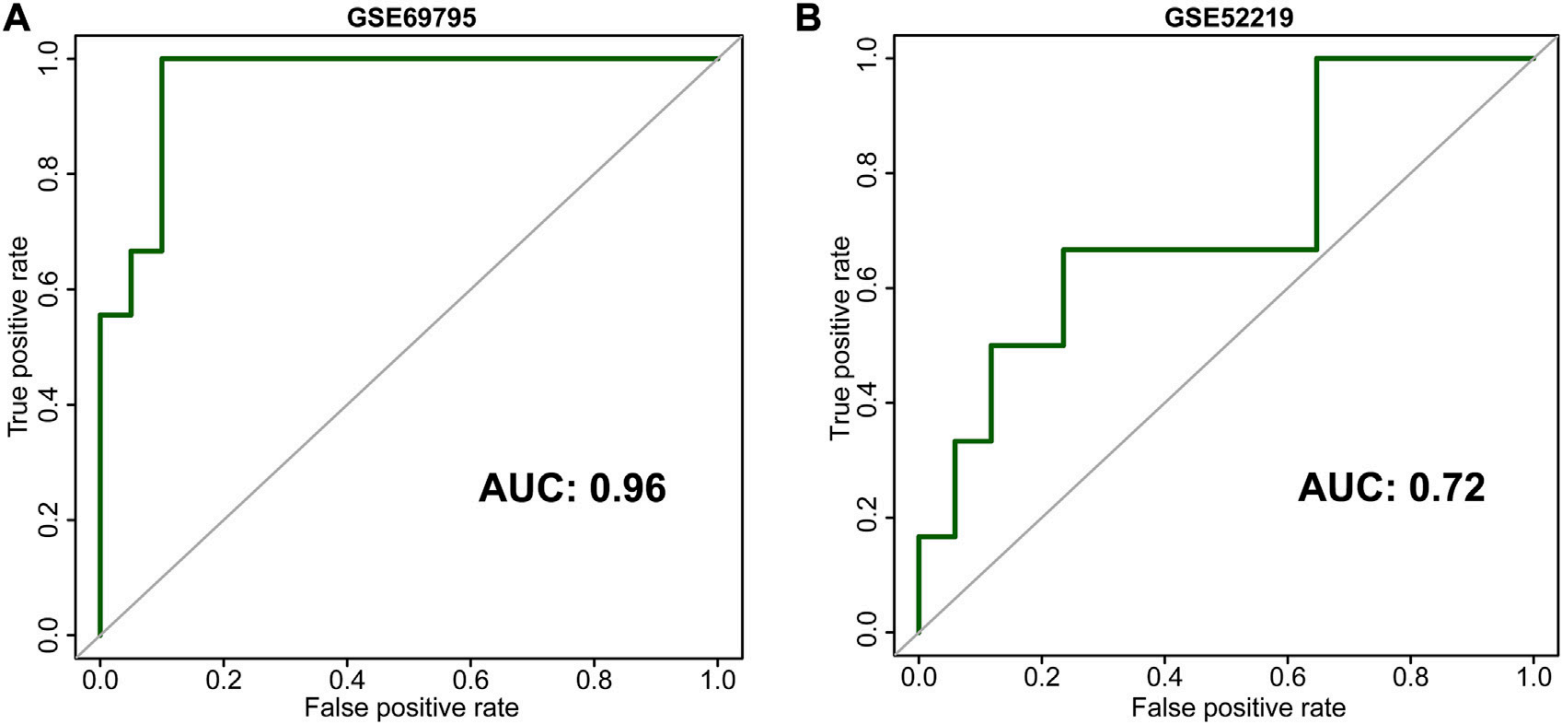
Receiver operating characteristic curves (ROCs) of our development [**(A)**, GSE69795] and validation [**(B)**, GSE52219] cohorts.

**FIGURE 3:**
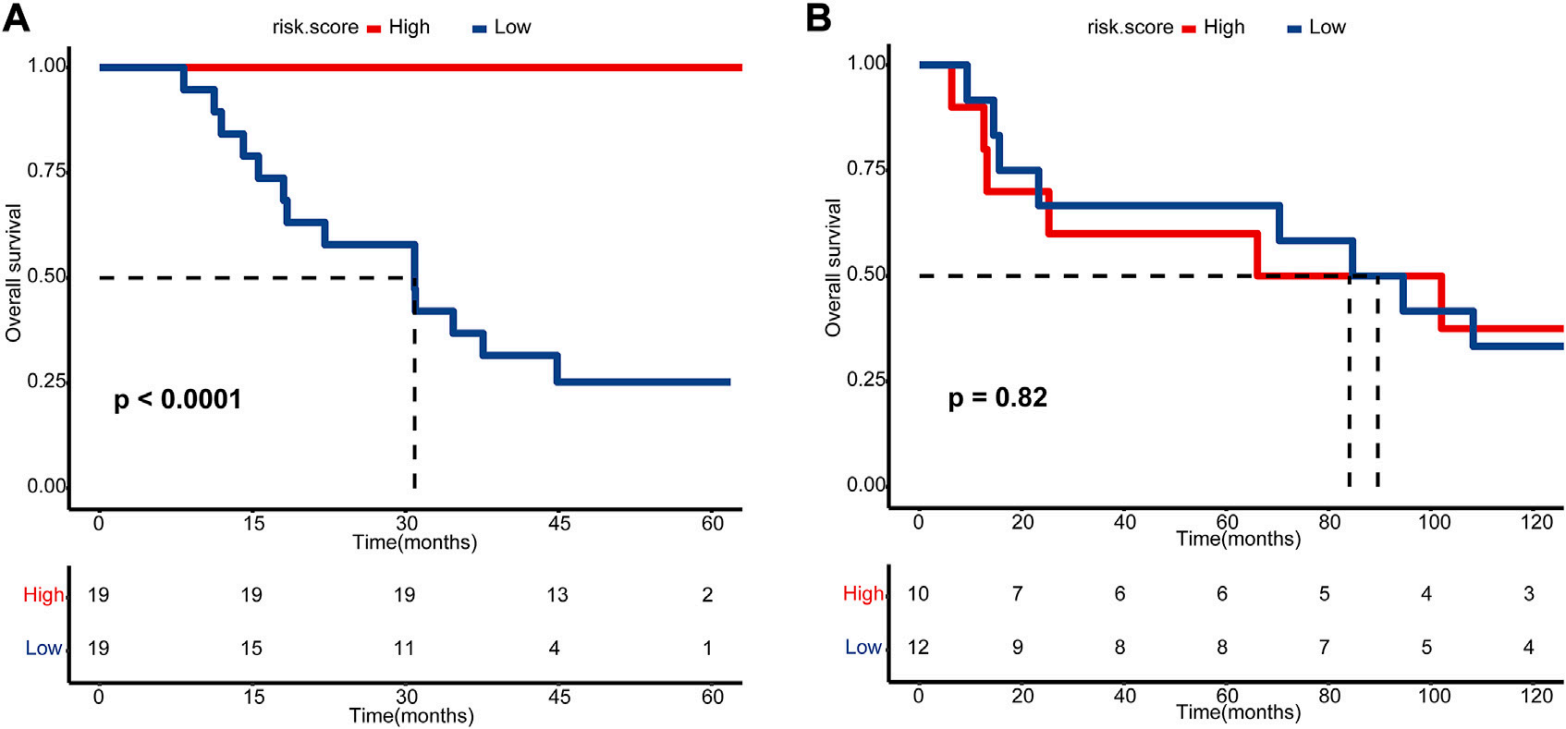
Kaplan–Meier estimated plot comparing the overall survival of patients with high- and low-risk score groups. **(A)** GSE69795 and **(B)** GSE52219. The red line represents the high-risk score group, while the blue line represents the low-risk score group.

Microarray data on 38 patients from GSE69795 were used to build a gene coexpression network to find the module with the closest relationship to NAC response (Supplementary Figure S1A). Clinicopathologic variables from GSE69795 included age, PS score, lymphovascular infiltration (LVI), clinical stage, and NAC response (Supplementary Figure S1A). Setting the scale-free *R* ^2^ as 0.85, we selected 6 as the soft threshold value to build the scale-free network (Supplementary Figure S1B). Twenty-nine modules were identified, and the green module had the closest relationship (R = 0.36, *p* = 0.02) with NAC response (Supplementary Figures S1C,D). There were 1819 genes in the green module, and all the genes were significantly coexpressed (cor = 0.45, *p* < 0.001, Supplementary Figure S1E). Using the LASSO algorithm, we identified five candidate genes, including *TMEM69*, *OR6W1P*, *CNNM1*, ***CEP83***, and *ACTC1*, according to lambda by one standard error (0.22) (Supplementary Figures S2A,B). An NAC-predicting risk score was then developed using a multivariable binary logistic regression model: risk score = 0.01*TMEM69 + 2.25*OR6W1P-0.67*CNNM1+5.68*CEP83-0.21*ACTC1. A higher risk score was associated with a higher NAC response rate. The predictive accuracy was extremely high in the GSE69795 cohort, with an area under the curve (AUC) index of 0.96 (Figure 2A). Importantly, this risk score could be validated in GSE52219 (AUC: 0.72, Figure 2B), indicating satisfactory predictive accuracy. In addition, this risk score could also predict the OS of patients in GSE69769 (*p* < 0.0001, Figure 3A), although this value was not statistically significant in GSE52219 partly because of the small sample size (*p* = 0.82, Figure 3B).

**FIGURE 4:**
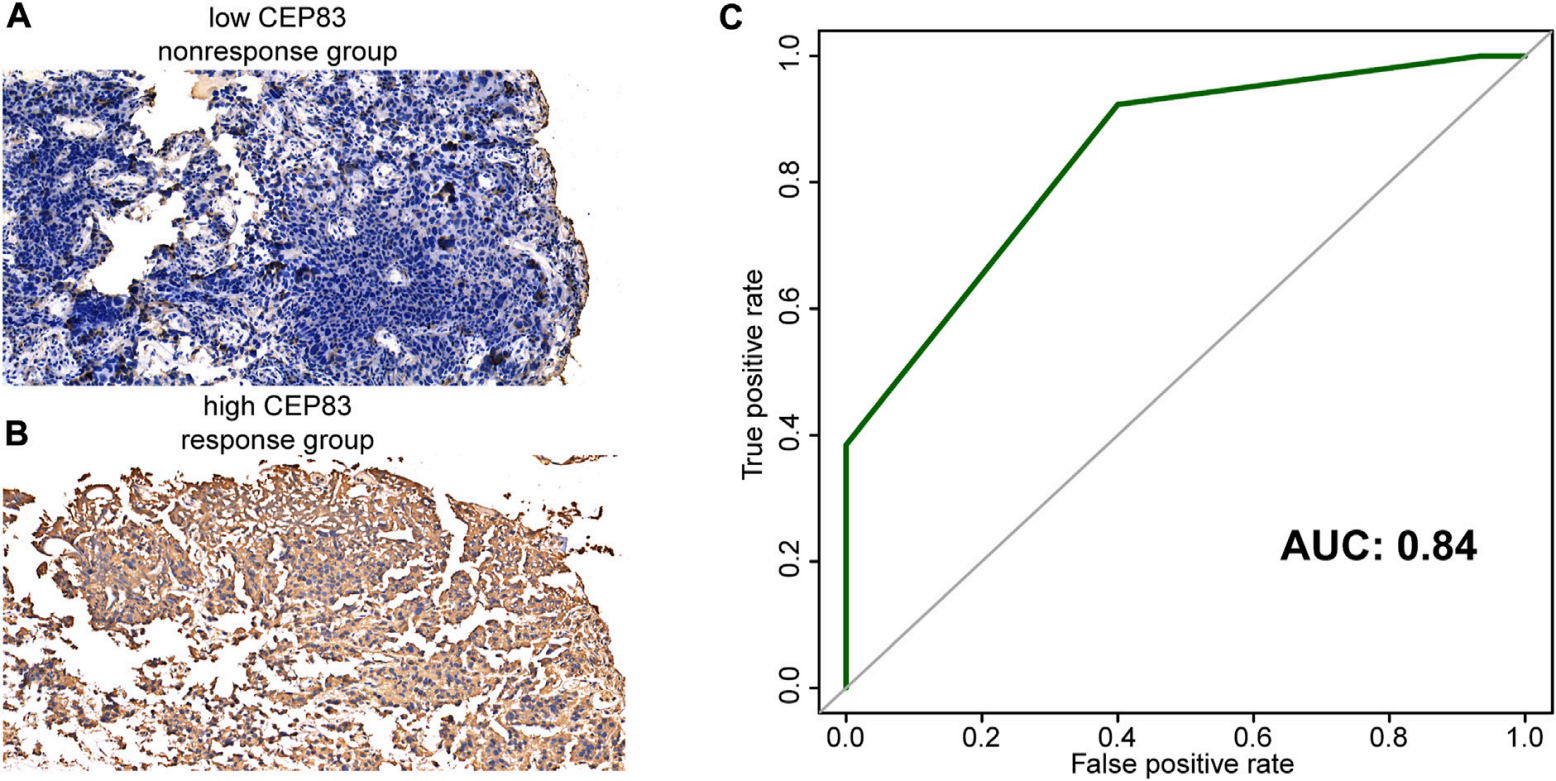
Representative images of CEP83 staining. **(A)** Negative staining (no response group). **(B)** Positive staining (response group). **(C)** Receiver operating characteristic curves (ROCs) of CEP83 in our IHC cohort.

As *CEP83* possessed the highest β value in this risk score, we further evaluated the association between NAC response and CEP83 only. To our surprise, *CEP83* possessed relatively satisfactory predictive accuracy for NAC response, with AUCs of 0.84 and 0.63 in GSE69795 (Supplementary Figure S3A) and GSE52219 (Supplementary Figure S3B), respectively. We collected 28 pretreatment FFPE samples and validated the predictive value of *CEP83* using IHC. Figures 4A,B show representative images of *CEP83* staining. The predictive value of *CEP83* for NAC response was successfully validated in our own IHC cohort (AUC: 0.84, Figure 4C). This result could largely simplify the predictive system for NAC.

## Discussion

For the treatment of MIBC, RC and lymphadenectomy have remained the gold standard for the last 3 decades. Since 1999, the Medical Research Council (MRC) has performed a randomized phase III trial, comparing the efficacy of cystectomy and/or radiotherapy with or without three prior cycles of cisplatin, methotrexate, and vinblastine (CMV). This trial concluded that NAC could contribute to a significant 16% reduction in death for patients with MIBC (International collaboration of trialists, 1999; Griffiths et al., 2011). Meanwhile, the Southwest Oncology Group (SWOG)-8710 trial demonstrated that NAC therapy combined with methotrexate, vinblastine, doxorubicin, and cisplatin (MVAC) was associated with a significantly improved survival benefit (Grossman et al., 2003). Based on these two RCTs, NAC plus RC is becoming the standard treatment option for MIBC. The underlying mechanism was that NAC could eradicate micrometastatic disease and downstage the primary MIBC tumor (Buttigliero et al., 2017). However, despite this level 1 evidence, the actual adoption of NAC for MIBC is slow and unsatisfactory, with only 1.4%–20.9% of patients receiving NAC even in contemporary cohorts (Keegan et al., 2014; Kim et al., 2015; Reardon et al., 2015). The proportions of elderly patients, poor PS, multiple comorbidities, and renal insufficiency could be the reasons for the low utilization of NAC (Anan et al., 2017), and patients with these features might be excluded from RCT trials. Therefore, we conducted this research to summarize the real-world practice of NAC in China for the first time. Although we set no strict rules for patient inclusion, all the patients we included were in good PS with an ECOG score of 0 or 1. For the fear of renal insufficiency, only two patients (3%) received an NAC program of gemcitabine and carboplatin because of renal insufficiency. Both patients remained muscle invasive in RC, indicating the lower effectiveness of carboplatin. In addition, the side effects of NAC were acceptable, with only two grade Ⅳ and seven grade Ⅲ AEs reported. No treatment-related deaths or delays in surgery proved that NAC was relatively safe in the real-world practice. Unfortunately, only a small portion (46%, 23/50) of patients achieved a pathologic response from NAC. Additionally, 16 patients (24.2%, 16/66) gave up radical treatment because of economic reasons or a lack of confidence in curing. These two facts again emphasize the importance of NAC as a predictive biomarker.

Some studies reported an association between clinicopathologic variables and NAC response. Boeri et al. reported that current smokers and previous smokers had four and two times the risk of non-response to NAC, respectively (Boeri et al., 2019). However, Kim et al. concluded that there was no relationship between smoking and NAC response (Kim et al., 2014). For urothelial carcinoma mixed with other tumors, Kaimakliotis et al. reported that NAC could contribute to equal oncological outcomes for patients with pure urothelial carcinoma or mixed tumors (Kaimakliotis et al., 2016). Unfortunately, we could only determine that there was no difference in the pathological type between responders and non-responders and could not calculate the relationship between the pathological type and NAC response because of the small sample size. Interestingly, a high neutrophil-to-lymphocyte ratio (NLR) was related to worse OS and poorer response to NAC (Gondo et al., 2012; Buisan et al., 2017). We did not draw this conclusion in our study, and this may need further research in the future. Lyon et al. stratified patients receiving NAC into low- and high-risk groups based on clinicopathologic variables including hydronephrosis, LVI, variant histology, and clinical T stage (Lyon et al., 2019). In our study, we found that higher tumor grade and clinical stage were both significantly associated with NAC non-response, indicating that tumor grade might also be included as a variable factor for stratifying patients in the future. Although fewer patients with a higher clinical T stage (cT3 or cT4) achieved a pathologic response, we should not exclude them from lifesaving NAC as RC alone cannot cure them because of extravesical invasion. Therefore, we further explored the gene-based predicting signature for NAC.

The major mechanism of cisplatin is to cause DNA damage, affect cell survival, and induce apoptosis. As a result, the association between mutation of DDR genes and response to NAC is widely reported (Motterle et al., 2020). Plimack et al. reported that genomic mutations of ATM, RB1, and FANCC were associated with response and OS after cisplatin-based NAC (Plimack et al., 2015). Somatic mutation of *ERCC2*, another DDR gene, was found to be correlated with a complete pathologic response in the study of Van Allen et al. (2014) and validated in Liu et al. (2016). However, another study found that the association between ERCC2 missense mutations and pathologic response did not reach statistical significance, while only ERBB2 mutations were significantly related to response (Groenendijk et al., 2016). Another focus is molecular subtypes and NAC response. Patients with MIBC can be generally divided into basal/squamous (characterized by squamous features) and luminal (characterized by papillary features) subtypes based on gene expression profiling (Motterle et al., 2020). Choi et al. divided patients into basal, luminal, and p53-like subtypes and found that basal tumors are characterized by aggressiveness and a better response to NAC, while p53-like tumors are characterized by resistance to NAC (Choi et al., 2014). However, all these reports have not translated into clinical applications partly because of inconsistent results, unsatisfactory predictive accuracy, or complex detection methods. Based on the gene expression profiles of 27 patients with MIBC, Takata et al. developed and validated a 14-gene-based signature for NAC prediction (Takata et al., 2005). In addition, Kato et al. developed a signature for accurate NAC prediction consisting of 12 “predictive” genes (Kato et al., 2011). In our study, we developed (38 patients) and validated (23 patients) a five-gene-based gene signature for extremely accurate prediction with AUC scores of 0.96 and 0.72, respectively. For routine clinical application, detection of a five-gene-based signature is much easier and convenient for MIBC patients planning to receive NAC therapy, and our findings led to the achievement of “personalized therapy”. Solmi et al. reported for the first time that TMEM69 could be a biomarker for the metastasis of colon cancer (Solmi et al., 2006). Cyclin M (CNNM) families were vital factors in maintaining cellular and body magnesium (Mg^2+^) homeostasis (Giménez-Mascarell et al., 2019). For CNNM1, it was reported that CNNM1 could be regulated by SNHG7 and miR-9-5p to promote the progression of hepatocellular carcinoma (Xie et al., 2020). Moreover, ACTC1 was reported to be a novel marker for prognosis of bladder cancer (Liu et al., 2021). We further narrowed down this five-gene-based signature to *CEP83* for convenient clinical application. As a protein-coding gene, *CEP83* was reported to be associated with nephronophthisis, and we found it was related to NAC response for the first time (Failler et al., 2014).

Limitations to our study: First, this is a retrospective study with a small sample size, and there were some patients with missing data. It is impossible to carry out some deep analyses, such as subgroup survival analysis, and this fact may indicate bias in our study. However, to the best of our knowledge, this is the first real-world NAC practice from China, and our study has a complementary effect on RCTs. Second, although our risk score exhibited an accurate predictive value for NAC in the development and validation cohorts, its predictive value should be further validated in larger prospective clinical trials.

## Conclusion

NAC was relatively safe and could significantly improve the overall survival for MIBC patients in the real-world practice. Our five-gene-based risk score could guide personalized therapy and promote the application of NAC. The *CEP83* expression exhibited an extremely important value for NAC response prediction.

## Data Availability

The original contributions presented in the study are included in the article/Supplementary Material; further inquiries can be directed to the corresponding author.
