# Supplementary material for: A novel signature to predict the neoadjuvant chemotherapy response of bladder carcinoma: Results from a territory multicenter real-world study
Source: Front Genet. 2022 Nov 2;13:1047481. doi: 10.3389/fgene.2022.1047481 (PMC9667090; doi:10.3389/fgene.2022.1047481)
Supplement: Supplementary file 3 [file Table1.docx]

**Table S1.** The clinicopathologic variables of all the patients included in our study.

| **Age** (years), median (range) | 61.3 (30-80) |
| --- | --- |
| **Gender**, n (%) |  |
| Male | 60 (87.0%) |
| Female | 9 (13.0%) |
| **ECOG status**, n (%) |  |
| 0 | 58 (84.1%) |
| 1 | 11 (15.9%) |
| **BMI** (kg/m^2^), median (range) | 22.2 (15.2-28.9) |
| **Smoking status**, n (%) |  |
| Never | 28 (40.6%) |
| Former | 10 (14.5%) |
| Current | 31 (44.9%) |
| **Hematuresis,** n (%) |  |
| No | 7 (10.1%) |
| Yes | 62 (89.9%) |
| **History of NMIBC,** n (%) |  |
| No | 55 (79.7%) |
| Yes | 14 (20.3%) |
| **Previous intravesical instillation,** n (%) |  |
| No | 57 (82.6%) |
| Yes | 12 (17.4%) |
| **Hydronephrosis,** n (%) |  |
| No | 45 (65.2%) |
| Yes | 24 (34.8%) |
| **Pathological type,** n (%) |  |
| Pure urothelial carcinoma | 64 (92.8%) |
| Mixed tumors | 5 (7.25%) |
| **Pathological grade** |  |
| Low | 17 (24.6%) |
| High | 52 (75.4%) |
| **T stage** |  |
| T2 | 20 (29.0%) |
| T3 | 29 (42.0%) |
| T4 | 20 (29.0%) |
| **N stage** |  |
| N0 | 58 (84.1%) |
| N+ | 11 (15.9%) |
| **NAC program** |  |
| Gemcitabine + cisplatin | 67 (97.1%) |
| Gemcitabine + carboplatin | 2 (2.90%) |
| **Surgery type** |  |
| RC | 44 (63.8%) |
| TURBT | 8 (11.6%) |
| Partial cystectomy | 1 (1.45%) |
| None | 16 (23.2%) |
