# Supplementary material for: A novel signature to predict the neoadjuvant chemotherapy response of bladder carcinoma: Results from a territory multicenter real-world study
Source: Front Genet. 2022 Nov 2;13:1047481. doi: 10.3389/fgene.2022.1047481 (PMC9667090; doi:10.3389/fgene.2022.1047481)
Supplement: Supplementary file 4 [file Table2.docx]

**Table S2.** Summarized adverse events (AEs) related to neoadjuvant chemotherapy (NAC) of all the patients included.

| **ADVERSE EVENTS** |  | **Grade** | | | |
| --- | --- | --- | --- | --- | --- |
|  | 0 | Ⅰ | Ⅱ | Ⅲ | Ⅳ |
| Anemia | 27 | 22 | 13 | 5 | 2 |
| White blood cell decreased | 42 | 18 | 7 | 2 | 0 |
| Neutrophil count decreased | 63 | 4 | 2 | 0 | 0 |
| Platelet count decreased | 65 | 4 | 0 | 0 | 0 |
| Hemorrhage | 64 | 5 | 0 | 0 | 0 |
| Bilirubin increased | 57 | 12 | 0 | 0 | 0 |
| Alanine aminotransferase increased | 64 | 5 | 0 | 0 | 0 |
| Alkaline phosphatase increased | 63 | 5 | 1 | 0 | 0 |
| Mucositis oral | 64 | 4 | 1 | 0 | 0 |
| Vomiting | 60 | 9 | 0 | 0 | 0 |
| Diarrhea | 62 | 7 | 0 | 0 | 0 |
| Creatinine increased | 49 | 19 | 1 | 0 | 0 |
| Fever | 67 | 2 | 0 | 0 | 0 |
| Alopecia | 59 | 9 | 1 | 0 | 0 |
| Cardiac rate | 62 | 5 | 2 | 0 | 0 |
| Constipation | 61 | 8 | 0 | 0 | 0 |
